# Informed Written Consent for Emergency and Elective General Surgery at a Model 4 Hospital: A Closed-Loop Audit

**DOI:** 10.7759/cureus.49480

**Published:** 2023-11-27

**Authors:** Ke En Oh, Nikhil Vasandani, Afiq Anwar, Babak Meshkat

**Affiliations:** 1 Department of Surgery, University Hospital Galway, Galway, IRL; 2 Department of Surgery, Royal College of Surgeons in Ireland, Dublin, IRL

**Keywords:** health professional's education, general trauma surgery, git endoscopy, clinical audit system, quality improvement projects, surgery general

## Abstract

Introduction

The objective of this investigation was to conduct an audit of the consent form standards signed by patients before elective or emergency general surgery at our institution. The investigation involved a comparison of these standards with those outlined in the "HSE National Consent Policy 2022" established by the Health Service Executive (HSE) and the Royal College of Surgeons in Ireland (RCSI). In the event of discrepancies, we intended to complete the audit loop by educating general surgeons on the essential standards for obtaining written consent in both elective and emergency general surgical procedures.

Methods

To assess the quality of patient consent, a pre-interventional phase was conducted over one week. Information was gathered exclusively through electronic medical record systems. Subsequent to the data analysis, an in-person educational session was conducted to enlighten non-consultant hospital doctors (NCHDs) in surgery about the significance of informed written consent and the criteria for lawful consent according to local guidelines established by the HSE and the RCSI. Three months following the intervention, a follow-up cycle was carried out to evaluate whether there were any improvements in the standards of consent.

Results

In the initial phase, prior to intervention, a total of 95 consent forms were collected. The patient's name, date of birth (DOB), and hospital board number (BN) were accurately recorded in all consent forms. However, only 66% (n=63) were accurately documented without the use of abbreviations or acronyms. Following the intervention, 145 consent forms were gathered. All appropriately indicated the patient's name, DOB, and BN. However, 84% (n=122) of consent forms were correctly labeled without the use of abbreviations or acronyms (p=0.0017).

Conclusion

This closed-loop review illustrates that the quality of consent can be notably enhanced through a straightforward educational intervention led by NCHDs in general surgery. Such interventions can be instructive, leading to improved consent form documentation. This, in turn, enhances patient safety and helps prevent potential medico-legal repercussions for both healthcare providers and institutions.

## Introduction

Informed written consent is an important aspect of perioperative care. It is an ethical and legal requirement that all healthcare professionals must obtain prior to performing surgery [1]. In recent decades, the medical community has become increasingly concerned about inadequate documentation and inappropriate consent practices prior to surgical intervention [2]. Given that Ireland, unfortunately, boasts one of the greatest medical litigation rates in Europe, it is plausible to assert that insufficient consent may give rise to medical malpractice claims and legal controversies. These, in turn, have the potential to lead to financial repercussions, adverse public perception, and emotional distress for all parties involved, including the patient, healthcare provider, and the institution. To address these concerns, healthcare organizations should prioritize the development and implementation of comprehensive consent processes that protect patient rights and minimize risks associated with consent. Some studies have emphasized the importance of standardized consent forms to facilitate consultation and structured pre-operative documentation, while others have recommended implementing written consent forms for surgery to reduce potential medico-legal disputes [3-5].

Referrals to the general surgery service represent a substantial portion of patients seeking care in our teaching hospital. Within these referrals, a significant number of patients require operative interventions for various surgical conditions either in the form of emergency or elective procedures. Prior to surgery, obtaining informed consent is an absolute necessity for surgical candidates. While informed consent in various contexts might be acquired verbally or implicitly, surgical procedures necessitate written consent, incorporating crucial elements to validate its legitimacy. These elements encompass, among others, the patient's name, date of birth (DOB), hospital board number (BN), and the specific operation, all presented without abbreviations or acronyms [6]. Nevertheless, a comprehensive examination of existing literature indicates a noteworthy prevalence of inaccurately completed consent forms in general surgical operating theaters, often attributed to errors made by general surgeons [7].

Therefore, it is hypothesized that the fundamental components essential for achieving optimal informed consent are absent from the consent forms signed by patients undergoing general surgery at our hospital. This study aims to conduct an audit of the standards present in the consent forms signed by our patients before undergoing emergency or elective general surgical procedures. This was compared with the standards outlined in the "HSE National Consent Policy 2022," established by the Health Service Executive, the health division of the Government of Ireland, and the "Code of Practice for Surgeons" standards set by the Royal College of Surgeons in Ireland, the national authority overseeing surgical practice and training in the Republic [8,9]. If necessary, the objective would involve completing the audit cycle by educating general surgical consultants and non-consultant hospital doctors (NCHDs) regarding the essential standards for obtaining written informed consent. Our main hypothesis suggests that, in the initial audit cycle, approximately half of the consent forms would lack a fundamental element of informed consent. Our secondary hypothesis anticipates that implementing a simple educational intervention to raise awareness among surgeons would significantly improve the quality of consent forms collected.

## Materials and methods

This audit was approved by the Galway University Hospital Clinical Audit Committee on October 5, 2022 (Audit Number 303, 2022) under the title "Informed Written Consent for General Surgery at a Tertiary Referral Centre." We conducted a retrospective review of patients undergoing both elective and emergency general surgical procedures at our hospital. The review period lasted one week from July 11, 2022 to July 18, 2022. To assess the quality standard of the consent forms, two independent assessors (KO and NV) accessed the electronic medical records of patients who underwent surgery over the mentioned time period. The data collected underwent internal validation by a third reviewer (AA).

The main outcomes included: the name of the patient undergoing intervention, the patient's date of birth (DOB), the patient's hospital identification or BN, and the name of the planned procedure written in a non-abbreviated manner. For example, general terms like "EUA" must be listed as "examination under anesthesia," "lap" must be labeled as "laparoscopic," and "WLE" must be written as "wide local excision" to name a few.

Following the initial analysis conducted by the principal investigators in collaboration with the project supervisor, it was determined that an additional audit cycle would be necessary following the intervention to further assess the efficacy of the intervention on consent standards. An in-person educational approach to raise awareness of the importance of the consent process was held during an interdepartmental journal club which usually receives attendance from NCHDs and consultants in the department of general surgery. This was advertised as a mandatory session and usually receives good attendance. A further audit cycle was performed prospectively to close the loop. This would assess the impact of the intervention by reviewing consent forms completed from October 24, 2022 to November 7, 2022.

A statistical analysis was performed using GraphPad Prism 9.5.1 (GraphPad Software, San Diego, United States). Fisher’s exact test was carried out to determine whether the data obtained from both cycles were statistically significant. A p-value of less than 0.05 was considered to be statistically significant. It is important to note that since this audit solely pertained to assessing standards without the collection or analysis of clinical data, ethical considerations did not necessitate seeking informed consent from patients before the commencement of the audit.

## Results

Pre-intervention cycle

After completing the initial assessment cycle, it was determined that a total of 95 patients underwent emergency or elective general surgical procedures at our institution during the aforementioned time period. All 95 pre-intervention consent forms were analyzed. About 51% of all patients included were female with the rest being male. It was found that all consent forms accurately included the patient's name, date of birth, and board number. However, only 63 forms (66%) were filled out without the use of any abbreviations or acronyms.

Post-intervention cycle

Following the proposed in-person educational session, a post-intervention analysis was performed on the general surgical procedures in our hospital after three months. About 145 patients received surgical management during this period. Amongst these, 58% were female with the rest being male. Consent forms were analyzed for all 145 patients and compared with the consent forms analyzed from the pre-intervention cycle. It was observed that every consent form accurately recorded the patient's name, DOB, and hospital board number. About 84% (n=122) of all consent forms were accurately filled out without the use of abbreviations or acronyms. This demonstrated an improvement of 18% compared to the pre-intervention cycle (p=0.0017). Results from both cycles are presented in Table 1.

**Table 1:** Results from pre- and post-intervention cycles

	Pre-intervention	Post-intervention	p-value
Total number of patients	95	145	-
Name of patients	95 (100%)	145 (100%)	>0.99
Date of birth	95 (100%)	145 (100%)	>0.99
Hospital board number	95 (100%)	145 (100%)	>0.99
Acronym/abbreviation	63 (66%)	122 (84%)	0.0017

## Discussion

The most important discovery that can be extrapolated from this closed-loop audit was that the quality of informed written consent can be massively improved via a simple educational measure. This improvement can be achieved through a straightforward educational intervention designed to heighten awareness regarding the precise execution of informed written consent and adherence to prevailing national policies and medico-legal regulations. These uncomplicated educational sessions hold promise for augmenting the documentation of planned general surgical procedures, ultimately contributing to the optimization of patient safety.

The practice of organizing seminars to educate doctors on obtaining appropriate consent is not a novel approach. The Agency for Healthcare Research and Quality has also emphasized the importance of promoting awareness of correct consenting standards among healthcare professionals to ensure legal validity. One method they endorse is the implementation of interactive workshops and/or online training sessions to guide healthcare professionals in the informed consent and authorization process. Additionally, they recommend engaging experts in health literacy and informed consent to educate healthcare professionals and facilitating forums to discuss strategies for identifying and addressing liability concerns in the informed consent and authorization process. These approaches are practical, attainable, and, in a broader context, easily implementable by well-supported institutions, especially large model 4 hospitals [10].

Furthermore, a prevalent issue within the Irish healthcare system involves junior doctors independently obtaining consent from patients for complex procedures on a regular basis. Many of these doctors lack the requisite knowledge concerning common and severe procedural risks or complications. A recent study conducted by Rohan et al. disclosed that 83.6% of intern doctors, in their first year of postgraduate training, reported that their supervisors did not provide explanations of procedures before obtaining consent. Of those surveyed, 92.4% admitted to consenting for procedures they had never witnessed, and 35.1% acknowledged consenting for procedures without thoroughly discussing the surgery and associated risks [11]. This practice poses significant risks as it hampers proper informed consent, potentially leading to medico-legal repercussions that could jeopardize a career. While it is recognized that intern doctors are compelled by significant resource limitations due to the shortage of doctors in Ireland, it is believed that this issue can be addressed through simple educational interventions aimed at raising awareness of best-consenting practices among this cohort.

In the perioperative setting, obtaining informed consent remains a challenging aspect of hospital-level patient care, influenced by factors such as the readability of consent forms, the patient's reading level, and perception [12]. Heterogeneity among Irish hospitals has been observed regarding consent forms, with the majority using single forms, highlighting the need for standardization to minimize litigation risk for doctors [13]. In addition, the current understaffing of healthcare workers in public Irish institutions indirectly plays a contributory role in this debacle. Abbreviations are often used to save time and space when writing in patient’s medical records [14]. In most circumstances, a single junior doctor is responsible for consenting all patients undergoing low and moderate-risk procedures, while consenting for high-risk interventions is supposed to be reserved for more senior surgeons. Regrettably, the actual practice often deviates from the recommended approach, as previously discussed [11]. The monotony of a single doctor having to sequentially consent, admit, and perform the necessary pre-operative requirements for 20 patients in a row for an elective list tends to encourage the use of abbreviations and acronyms as a means to expedite the consent process. This was the rationale provided by most doctors when informally interviewed as part of this study.

Although guidelines emphasize the importance of clear, concise, and non-abbreviated consent forms, standardization remains a challenge [15,16]. Standardized forms have been shown to be easier to read and understand. Standardized forms are important in ensuring consistency of documentation which in turn creates uniformity in practice [12]. Currently, in Ireland, there is a high turnover in university teaching hospitals with NCHD staff rotating through different hospitals annually. This makes it difficult to establish a formal culture of consenting but also highlights the urgent need for standardized consenting as a method to resolve this issue [16]. At the moment, no standardized consent forms for elective and emergency general surgical procedures exist within the Republic. The idea of standardized consent forms has been proposed for elective general procedures, but are not applicable in the very diverse and unique context of general surgical cases [17].

It is proven by this study that education plays an important role in the consent process. Online and traditional teaching sessions have shown great promise in improving patient recall of consent and consent procedures [16]. However, data specific to general surgeons obtaining written informed consent are lacking. Research shows that virtual education sessions, not only for patients but also for surgeons, can improve the quality of written consent obtained. Therefore, providing virtual training sessions for patients and practitioners could optimize the quality of future written informed consent [18].

This is an excellent example of how an audit assisted our general surgery department in raising the quality of care to the level required by the National Health Service and surgical governing body. This quality improvement project has shown that reviewing practices on a regular basis can improve our service within a short time frame without the need for extensive resources and costs.

Limitations

While this closed-loop audit was successful in improving the standards, it is also important to recognize its limitations. First, the audit spanned a relatively brief period, encompassing only three months. Second, the audit cycles were conducted by a sole investigator, potentially introducing bias. Third, the data collection process was characterized by a binary approach, classifying errors as either correct or incorrect, thus neglecting a broader spectrum of potential variations. Finally, it was seen that even after the re-audit cycle, we still had a 16% failure rate. This suggests that the educational intervention proposed failed to target all medical staff it was intended for. This could be due to a small proportion of doctors failing to attend the educational session. Although it was advertised as mandatory, no formal system was in place to check attendance. A repeat session was not provided and no email communication was sent to target potential non-attendees or inattentive doctors. Utilizing email correspondence, posters, repeat sessions, and other forms of multimedia, including notifying doctor messaging groups via social media or instant messaging platforms, could have helped target this issue and enforce compliance.

In addition, looking back in retrospect, another limitation is that the level of training of each consenting surgeon was not evaluated. There was a lack of stratification as to the seniority of the physician taking consent which may be a cofounder. Generally, newly qualified junior doctors are more likely to be adherent to best-consenting practices relative to their more senior counterparts. Identifying the experience level of the consenter could help determine whether poor consenting practices are secondary to experience or reduced awareness. Furthermore, alternative modalities for delivering the intervention, such as a combination of in-person and virtual educational sessions, may be employed in subsequent studies to enhance awareness and promote adherence. Another major limitation of the project is that the study group was limited to a single general surgery department. As a result, the study’s findings may have constraints in generalizing to more diverse healthcare settings. Also, the study primarily focused on consent form documentation and neglected crucial aspects of the consent process, such as patient comprehension and satisfaction. This is a good parameter to target in future studies. A follow-up study to assess the enduring impact of the educational intervention on the quality of obtained consent needs to be performed. Despite this, the project fulfilled its goal of improving informed written consent practice in concordance with local guidelines.

## Conclusions

The findings from this closed-loop audit indicate that in-person educational sessions can significantly enhance the quality of informed consent obtained by general surgeons. These simple educational interventions have the potential to improve the documentation of elective and emergency surgical procedures for general surgical cases, thereby optimizing patient safety and avoiding potential medico-legal retributions impacting both surgeons and the institution. Given the high turnover of non-consultant hospital doctors within tertiary hospitals, regular training sessions on this topic can promote cultural change, help maintain high standards of documentation and informed consent for patients undergoing surgery.
